# Efficacy and safety of diflunisal therapy in patients with transthyretin cardiac amyloidosis (ATTR-CA): a systematic review and meta-analysis

**DOI:** 10.1186/s43044-025-00625-3

**Published:** 2025-03-11

**Authors:** Wilbert Huang, Alvin Frederich, Apridya Nurhafizah, Antania Devita Salma, Rivera Adenia Firza Zahrani, Intan Aulia Retnoningrum

**Affiliations:** Padjadjaran University, Bandung, Indonesia

**Keywords:** Transthyretin, Amyloidosis, Diflunisal

## Abstract

**Background:**

Transthyretin cardiac amyloidosis (ATTR-CA) is a progressive cause of diastolic heart failure associated with poor prognosis. Currently available treatment, tafamidis, a TTR stabilizer, is highly effective and tolerable but is not cost-effective. Hence, we aim to evaluate the efficacy and safety of a mechanistically similar but more affordable TTR stabilizer, diflunisal, in patients with ATTR-CA.

**Methods:**

Systematic searching until June 2024 was done on 3 databases to include patients with ATTR-CA of any type (hereditary or wild-type). Efficacy and safety of diflunisal are assessed by baseline to follow-up mean difference of specific clinical parameters and mortality risk reduction comparing intervention to the control group is evaluated by the generic inverse variance model. The proportion of discontinuation rate and adverse effects are evaluated with a single-arm inverse variance model. Statistical analyses are done with a random effect model conducted on RevMan and R software.

**Results:**

Twelve studies comprising 539 ATTR-CA patients with a mean of 70 years old are included. The majority of them are male with NYHA I–II severity and are being followed up for approximately 12 months. For diflunisal efficacy outcomes, we found no statistically significant changes in BNP, troponin I, LVEF, GLS, IVSD, PWD, and E wave from baseline to diflunisal posttreatment, however, we found a statistically significant posttreatment increase of transthyretin level (MD 9.34 mg/dL; CI 1.54–17.14; I^2^ 0%; *p* 0.02). We also found a statistically significant 77% (CI 58–87%; I^2^ 34%; *p* < 0.001) risk reduction of mortality in the diflunisal group compared to the control group. For diflunisal safety outcomes, we found a statistically significant reduction of eGFR, hemoglobin, and platelet count (MD − 5.55, − 0.32, − 11.61, respectively, *p* < 0.01) but no statistically significant change in creatinine level. Pooled proportions of discontinuation rate of diflunisal therapy is 24% (CI 15–36%; I^2^ 72%; *p* < 0.01) and adverse events causing therapy discontinuation are renal impairment (21%), GI impairment (13%), bleeding (6%), and fluid retention (6%).

**Conclusion:**

Diflunisal therapy is beneficial in treating ATTR-CA patients but is associated with adverse effects that require therapy discontinuation. Hence, careful monitoring during diflunisal therapy is necessary.

**Supplementary Information:**

The online version contains supplementary material available at 10.1186/s43044-025-00625-3.

## Introduction

Deposition of misfolded proteins in the form of amyloid fibrils into the myocardium leads to a condition called transthyretin cardiac amyloidosis (ATTR-CA), a slowly progressive yet underestimated cause of heart failure. ATTR account for approximately 13% case of heart failure with preserved ejection fraction that represents almost half of heart failure cases. Presence of cardiac amyloidosis with heart failure also increases the risk of death and hospital readmission by two and three times, respectively [1].

The growing increase in myocardial mass resulting from amyloid formation contributes to a smaller ventricular cavity size, causing diastolic filling abnormality as a common presentation. The ATTR-CA can be classified as either wild-type resulting from age-related failure (ATTRwt) or hereditary variant resulting from genetic mutation (ATTRv) [2, 3].

Currently, tafamidis is the first FDA approved transthyretin (TTR) stabilizer drug that has been proven to lower hospital admissions due to ATTR-CA. However, tafamidis use does not meet generally accepted cost-effectiveness threshold and their very high price impose important challenges associated with access and affordability that will affect patients, clinicians, and policymakers. Although associated with a favorable outcome and minimal side effects, the high cost issue has limited its use in clinical practice [4].

In the search for alternative therapies, diflunisal—a non-steroid anti-inflammatory (NSAID) agent— which also acts as TTR stabilizer could inhibit tetrameric TTR dissociation into unstable amyloidogenic monomers, can be an alternative agent to suppress amyloidogenesis [5]. Yet still, although the potential of diflunisal drug repurposing is intriguing, one must consider that chronic NSAID use has been associated with several adverse effects, notably GI impairment, renal dysfunction, fluid retention, furthermore increased morbidity and mortality in heart failure patients, creating hesitancy in prescribing a long-term regiment to cardiac amyloidosis patients. A clinically accepted dose of diflunisal, 250 mg orally twice daily, has been considered as an alternative in ATTR-CA treatment due to the similarity of mechanically and more affordable compared to the current first line therapy, tafamidis [6]. Hence, this potential advantage has led to an increase in our interest in evaluating the efficacy, yet still reflecting on possible adverse events of diflunisal as a viable and more affordable option for patients with ATTR-CA.

## Methods

### Search strategy

Systematic searching on three databases (PubMed, Scopus, Embase,) were done up until June 2024. Searching strategy mainly includes different terms associated with “transthyretin cardiac amyloidosis” and “diflunisal”. A detailed search strategy is elaborated in Supplementary 1.

### Study selection

Systematic study selection under prespecified inclusion and exclusion criteria was done by two reviewers independently (WH and AF). Any discrepancies or disagreements of study selection results were further discussed with the third author (AN). We specifically included both observational or randomized controlled trials with a population of amyloidosis transthyretin cardiac amyloidosis (ATTR-CA) undergoing diflunisal therapy. ATTR- CA diagnosis arising either from endomyocardial biopsy or consensus-agreed criteria were included. We also included both types of ATTR-CA namely, ATTR wild type (ATTRwt) and hereditary ATTR (hATTR). We excluded studies of review article, case report/ series, and animal studies. We also excluded studies in which intervention is described as TTR stabilizer only without any further details whether it includes tafamidis or diflunisal only therapy.

### Risk of bias assessment

The observational studies analyzed in this research were evaluated for risk of bias using the Revised Cochrane Risk of Bias Tool for Non-Randomized Studies of Interventions (ROBINS-I). This assessment considered various potential sources of bias, such as confounding factors, measurement of exposure, classification of interventions, participant selection, deviations from intended interventions, and missing data. Three reviewers (ADS, RAFZ, IAR) conducted the evaluations independently, resolving any disagreements through mutual consensus.

### Data extraction

Data from the included studies were retrieved by two independent reviewers (WH and AF). Two other reviewers (AN and ADS) evaluated all the collected data for any inconsistency and all authors came to a consensus to discuss any existing inconsistency. Data extracted from each included study include author, study name/center, period of study, study design, sample size, intervention used and its dose, concomitant treatment (such as proton pump inhibitors or 2nd generation antihistamine), population, types of ATTR-CA, genetic variance, baseline IVSD and ATTR level, anticoagulation use, and their outcomes.

### Outcome measures

Outcomes evaluated in this review include baseline and follow-up parameter changes of natriuretic peptide, troponin, TTR, left ventricular ejection fraction (LVEF), interventricular septal diameter (IVSD), posterior wall diameter (PWD), and global longitudinal strain (GLS) to assess the efficacy of intervention. We also evaluated binary outcomes of mortality in the intervention and control group. Additionally, we evaluated safety outcomes of changes in creatinine, estimated glomerular filtration rate (eGFR), hemoglobin, and platelet count from baseline to follow up. We also evaluated the proportion of discontinuation rate and the rationales behind the discontinuation (renal impairment, gastrointestinal impairment, bleeding, or fluid retention).

### Statistical analysis

Statistical analysis is conducted with ReviewManager version 5.4 and R studio software. Changes of parameters from baseline to follow up are measured as mean difference and pooled using inverse variance random effect models. Random effect model is chosen to address for various heterogenous result in each study. Missing data from studies are calculated accordingly based on Cochrane handbook guideline. Binary outcome comparing intervention and control group was pooled using inverse variance random effect model to generate pooled risk ratio. Proportions were pooled with inverse variance random effect model under logarithmic transformation. Confidence interval of 95% and *p* value < 0.05 were selected as statistical significance cutoff. Heterogeneity is being assessed by I^2^ with an interpretation of I^2^ < 40% as not important heterogeneity, 30–60% as moderate heterogeneity, 50–90% as substantial heterogeneity, and 75–100% as considerable heterogeneity. Publication bias is assessed with funnel plot for asymmetry.

## Results

### Study selection and characteristics

**Fig. 1:**
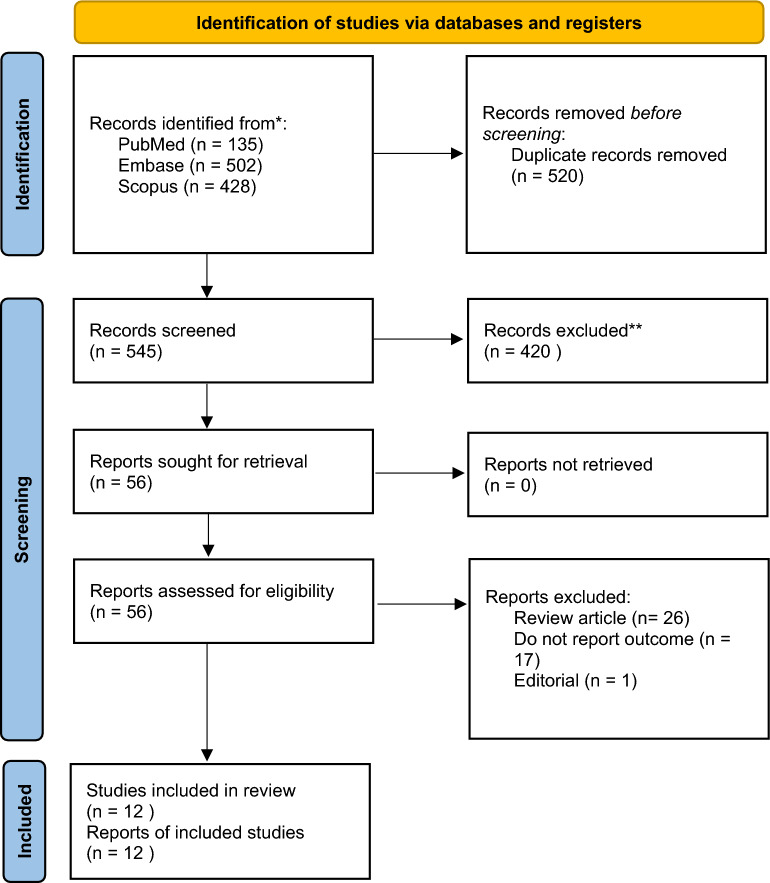
PRISMA flowchart diagram

Throughout the searching process, we identified a total of 1065 articles from three databases. After deduplication process, we obtained 545 articles. Further title and abstract screening resulted in 125 articles. After full text screening, 12 studies [7–18] are included in this systematic review and meta-analysis. (Fig. 1).

The included studies spanned between 2001 and 2023 with 11 (92%) studies are a retrospective cohort study, and one (8%) study is an open-label clinical trial study. These studies described a total sample of 895 subjects with either wild-type or hereditary ATTR-CA on which 452 subjects were administered diflunisal at some point of the respective study periods. All studies used the same twice a day 250 mg dosage regimen. Most of the subjects included were males and a mean age of 73.2 years old on all included subjects. Most of the samples included are a NYHA I-II (except in one study) with a normal LVEF (> 50%). The follow-up period ranges from 6 months to over 42 months. Outcomes assessed included a total of 9 parameters for efficacy outcome including: mortality rate, NT-pro BNP, troponin, TTR, Pre-albumin, LVEF, LAD, LVM, and GLS; and a total of 8 parameters of safety outcome including: creatinine, eGFR, hemoglobin, discontinuation rate, renal impairment, gastrointestinal toxicity, bleeding events, and fluid retention. (Table 1).

**Table 1:** Baseline characteristics of included studies

No	Author	Year	Country	Sample size (Intervention/Total)	Gender (Male)	Age (SD/IQR)	NYHA	Baseline level					Follow-up period
								LVEF (SD/IQR)	BNP (SD/IQR)	Troponin (SD/IQR)	IVSD (SD/IQR)	TTR (SD/IQR)	
1	Aventin	2018–2023	Spain	30/142	78%	78 (73–83)	97% NYHA I-II	60.1 (50.4–64)	848 (281–1279)	–	18 (16—19)	–	8,7 m
2	Castano	2009–2011	USA	13/13	85%	69 ± 3	69% NYHA I-II	50 ± 3	–	–	–	–	10,7 ± 3,4 m
3	Choi	2014–2021	Australia	61/136	–	–	–	–	–	–	–	–	6 m
4	Choi	2014–2021	Australia	108/108	–	–	–	–	–	–	–	–	6 m
5	Gilad	2009–2020	USA	43/66	98%	74 (68–78)	77% NYHA I-II	–	258 (148–449)	0.07 (0.05–0.11)	–	–	12 m
6	Hanson	2009–2016	USA	12/36	97.4%	76 (51–87.8)	81% NYHA ≥ II	50 (40–58)	373 (221–661)	0.114 (0.066—0.186)	15 (14—17)	23 mg/dL	12 m
7	Ikram	2007–2017	USA	23/23	87%	69.4 (64.1–75)	–	–	–	–	–	–	15 m
8	Koyoma	2003–2011	Japan	41/76	59%	57 ± 16	–	–	–	–	–	–	12 m
9	Lohrmann	2011–2016	USA	33/81	91.4%	74 (68–79)	–	–	–	–	–	21 (19–26)	12 m
10	Mints	2009–2016	USA	35/105	97%	76.7 (?)	–	–	–	–	–	–	12 m
11	Quarta	–	UK	18/35	–	70 (67—73)	–	I: 53 ± 9 C: 48 ± 10	(log) I: 5.9 ± 0.9 C: 6.2 ± 0.7	–	LV Wall Thickness: I: 16.6 ± 2 C: 16.9 ± 2	–	42 m
12	Sidiqqi	2009–2016	USA	35/104	97.1%	73.8 ± 7	82.8% NYHA I-II	53.1 ± 12	335 ± 365.6	0.1 ± 0.1	16.6 ± 2.7	–	12 m

Abbreviation: *NYHA*: new york heart association; *LVEF*: left ventricular ejection fraction; *BNP*: B-type natriuretic peptide; *IVSD*:interventricular septal end diastole; *TTR*: transthyretin

### Risk of bias assessment

The risk of bias assessment using the ROBINS-I protocol resulted in three studies (25%) being judged to have a moderate risk of bias and nine studies (75%) with a low risk of bias. A moderate risk of confounding bias was found in eight studies (66.67%). Bias in the classification of interventions was judged to be moderate in five studies (41.67%). Three studies (25%) were assumed to have a moderate risk of bias in the selection of participants. Bias due to deviations from intended interventions was moderate in one study (8.33%). Bias due to missing data was moderate in one study (8.33%) and low in the others (91.67%). The measurement of outcome bias was of moderate risk in two studies (16.67%).(Supplementary 2).

### Data synthesis

#### Efficacy of diflunisal therapy

**Fig. 2:**
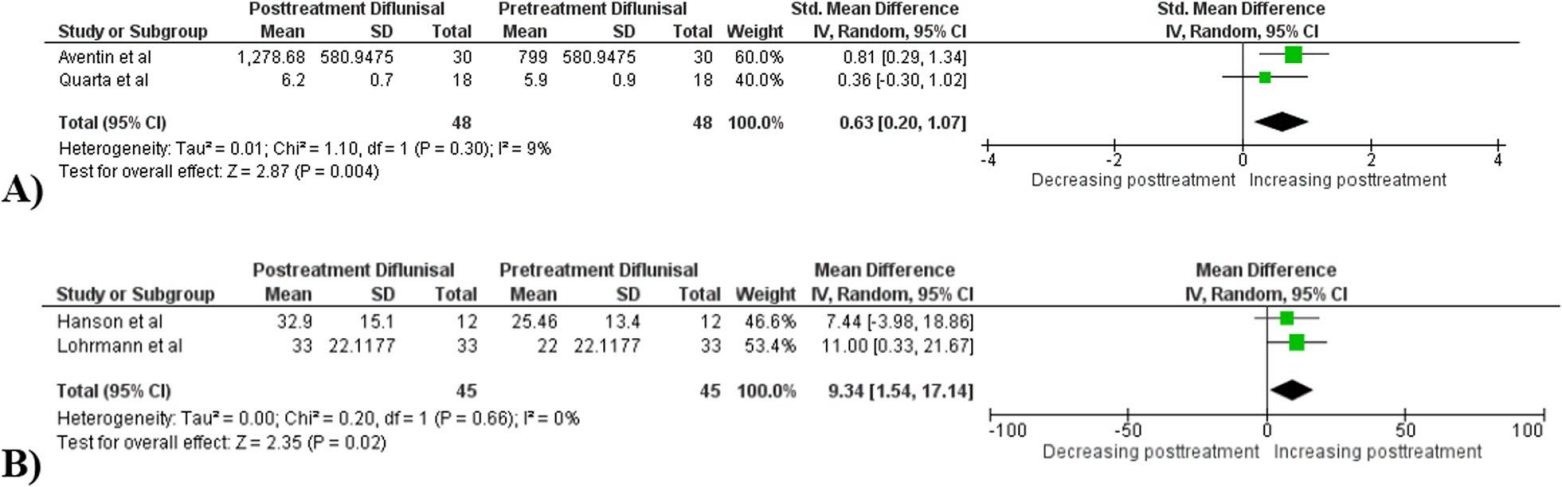
Posttreatment change of **A** NT- pro BNP—2 studies and **B** TTR level—2 studies with diflunisal therapy

Compared to baseline level, posttreatment with diflunisal therapy is not associated with a significant reduction of troponin I (4 studies, *p* = 0.59), BNP levels (6 studies, *p* = 0.57), PWD (4 studies, *p* = 0.84), E wave (2 studies, *p* = 0.79), and IVSD (5 studies, *p* = 0.12). In the pooled analysis of BNP outcome, we did not include Aventin et al. and Quarta et al. studies due to the different unit of measurement used in both studies (NT- pro BNP). (Supplementary 3–7) Separate analysis for NT- pro BNP outcome (2 studies, *p* = 0.36) however also did not result in a statistically significant reduction with diflunisal therapy. But when calculated as standardized mean difference, we found a statistically significant increase of NT-pro BNP level posttreatment with diflunisal therapy (SMD 0.63, CI: 0.20, 1.07, *p* 0.004, I^2^ 9%). (Fig. 2A) Additionally, posttreatment diflunisal therapy is also not associated with a significant increase of LVEF (6 studies, *p* = 0.40) and GLS level (4 studies, *p* = 0.86) compared to their baseline. (Supplementary 8–9) We found a statistically significant increase in TTR level posttreatment with diflunisal therapy with a mean difference of 9.34 mg/dL (CI 1.54–17.14; I^2^ 0%; p 0.02). (Fig. 2B) Changing the effect measure from mean difference to standardized mean difference did not result in changes in statistical significance nor changes in heterogeneity level except for NT-pro BNP outcome. After conducting leave one out sensitivity analysis in each outcome, we found that Castano et al. study resulted in larger change of heterogeneity level (I^2^) however, no change in statistical significance is found. Castano et al. study differs from the other in which it includes more patients with NYHA III/ IV severity (31% vs 3–23% in other studies).

**Fig. 3:**
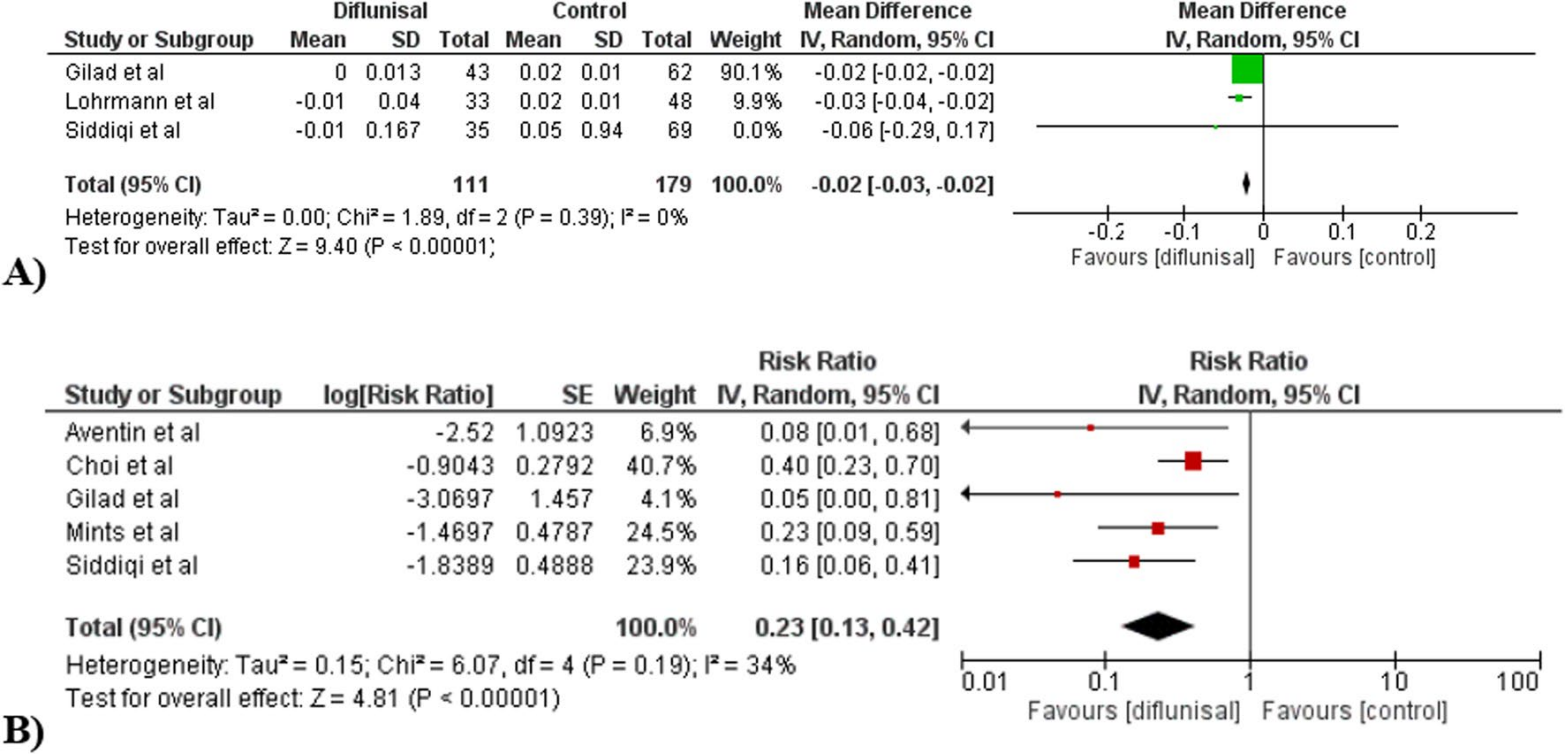
Changes of **A** troponin and **B** mortality outcome from 3 and 5 studies comparing diflunisal therapy with control

Compared to control group, we however found a statistically significant reduction in troponin level in diflunisal therapy group (MD − 0.02, CI: − 0.03, − 0.02, *p* < 0.00001, I^2^ 0%) (Fig. 3A). However, there are no significant changes other outcomes such as BNP (3 studies, *p* = 0.18), IVSD (4 studies, *p* = 0.24), E wave (2 studies, *p* = 0.35), PWD (3 studies, *p* = 0.49), LVEF (4 studies, *p* = 0.44), and GLS (4 studies, *p* = 0.40) when compared to control. (Supplementary 11–16).

Importantly, we found a statistically significant reduction of mortality in diflunisal group compared to control with RR 0.23 (CI 0.13–0.42; I^2^ 34%; *p* < 0.00001) (Fig. 3B). Subgroup analysis of only including studies within 12 months follow-up retains the statistically significant reduction of mortality (RR 0.18, CI: 0.09–0.34, *p* < 0.00001, I^2^ 0%). (Supplementary 17). Additionally, meta-analysis including only multivariate analysis studies also retain the statistical significance (RR 0.19, CI: 0.10–0.38; I^2^ 0%; *p* < 0.00001) (Supplementary 18). Leave-one-out analysis did not result in any statistically significant changes.

#### Safety of diflunisal therapy

For outcomes regarding safety concern, diflunisal is associated with a statistically significant posttreatment reduction of eGFR (MD − 5.55 ml/min/1.73 m^2^, CI − 9.75 − − 1.34, I^2^ 0%, *p* 0.01), hemoglobin (MD − 0.32 g/dL, CI − 0.57 − − 0.07, I^2^ 0%, *p* 0.01), and platelet count (MD − 11.61 × 10^3^/ µL, CI − 20.12, − 3.11, I^2^ 0%, *p* 0.007) compared to baseline. Although we found an increase of posttreatment creatinine level, it is not associated with statistical significance (4 studies, *p* = 0.07) (Supplementary 19–22).

Single arm meta-analysis of six studies showed that there was 24% (CI 15–36%, I^2^ 72%, *p* < 0.01) discontinuation rate of diflunisal use. Specifically, pooled analysis of three studies showed that 21% patients experienced renal impairment. We also found a 13% GI impairment adverse event in diflunisal therapy from meta-analysis of two studies in which all patients in Choi et al. uses proton pump inhibitor (PPI) while only 35% of patients in Ikram et al. uses PPI. There was a pooled 6% of bleeding events from three studies, and a pooled 6% of fluid retention from two studies. All of these reported outcomes resulted in discontinuation of diflunisal therapy. Leave-one-out analysis conducted on outcome of discontinuation rate shows that study by Choi et al. resulted in a change of heterogeneity level (I^2^ 72% to 28%) but not in the pooled proportion (24% to 21%).

## Discussions

In this meta-analysis, we found that diflunisal therapy did not result in significant changes of cardiac biomarkers (except for NT- pro BNP) and echocardiography parameters such as E wave, PWD, IVSD, and LVEF when compared to their baseline level. However, diflunisal therapy is associated with a significant increase of posttreatment TTR level. When compared to a control group, diflunisal therapy resulted in a significant reduction of troponin level but not with other parameters. Most importantly, diflunisal therapy is associated with a statistically significant reduction of mortality compared to the control group. For safety outcome, diflunisal therapy indeed resulted in a significant proportion of discontinuation rate mainly due to renal impairment.

Amyloidosis is an untreatable disease with poor prognosis depending on the types of amyloids and the genetic mutations affecting it. Generally, ATTR cardiac amyloidosis is a slowly progressive disease with a final common pathophysiologic pathway of diastolic and systolic cardiac dysfunction and the definitive therapy would be an orthotopic liver transplantation to replace mutant TTR to arrest amyloid formation [19, 20]. However, due to a shortage of liver donor, this is not widely available. Another option is to stabilize TTR amyloids with tafamidis, the only approved TTR stabilizer agent. However, due to the high cost of this agent, it is also not widely available.^4^ Hence, an alternative is to repurpose diflunisal, a non-steroidal anti-inflammatory drugs (NSAIDs), which has similar mechanism of action as tafamidis to stabilize ATTR amyloidosis patients [21].

Due to the poor prognosis of the disease with a median survival range of 3.5–6 years and limited therapeutic option [22], the main goal of current treatment is to prevent further progression of the disease by limiting the ATTR amyloid fibrillogenesis. In this meta-analysis, although we did not find a statistically significant improvement of cardiac parameters (except for NT- pro BNP), in parallel, we also showed that no disease progression was indicated based on these parameters with diflunisal therapy. Furthermore, we found a statically significant increase in TTR amyloids posttreatment with diflunisal therapy that indicates a good prognosis. Increase in TTR amyloids likely demonstrate an effective TTR stabilization and protection toward amyloid deposition in multiple organs. This is shown by a study by Hanson et al. [15] showing a halt of neurologic disease progression with a more stabilized TTR molecule in the circulation. Additionally, increase of serum TTR concentrations has also been shown to be a prognostic marker in ATTR cardiac amyloidosis patients [21].

In this meta-analysis, we also showed that diflunisal therapy when compared to a control group, resulted in a significant improvement of survival. Although not being able to have a side-by-side comparison with the currently approved agent, tafamidis, due to limited number of studies, this study demonstrated the potential of diflunisal as an alternative agent to treat ATTR cardiac amyloidosis. In a previous study by Elliot et al., the use of tafamidis is associated with a hazard ratio of 0.59 compared to placebo [23]. The lower hazard ratio in our study cannot be directly compared to the result from Elliot et al. due to the different types of study design used (randomized controlled trial) which is generally less susceptible to bias. Additionally, study by Damy et al. showed that treatment with tafamidis has similar incidence of adverse effects compared to placebo which indirectly emphasizes the safety of tafamidis [24].

As with other non-steroidal anti-inflammatory drugs, long-term use of diflunisal has been a concern. NSAIDs use have been associated with worsening renal function, gastrointestinal complications, and bleeding risks [25, 26]. Additionally, fluid retention associated with NSAIDs use could be detrimental in heart failure patients [27]. In our meta-analysis, we found a statistically significant reduction of eGFR, hemoglobin, and platelet count compared to their baseline levels. We also found a high discontinuation rate (24%) mostly due to renal impairment (21%), followed by GI impairment (13%). Additionally, 6% of the population develop fluid retention and bleeding events. These limitations of NSAIDs use are foreseeable risks of long-term use of NSAIDs and are inevitable. However, due to the potential survival benefit of diflunisal and no alternative agent, diflunisal should be considered for ATTR cardiac amyloidosis patient to halt disease progression. Current guideline has stated that NSAIDs increased morbidity and mortality in heart failure patients, however, when the use of NSAIDs is necessary, more rigorous awareness and more frequent follow-up are required in this high risk population [28].

The result from this meta-analysis could serve as a basis for hypothesis generation in future clinical trial studies addressing the non-inferiority of diflunisal compared to tafamidis in treating ATTR cardiac amyloidosis patients. This meta-analysis has also shown the potential survival benefit and adverse events of diflunisal therapy hence, future clinical decision can be made based on balancing the risk and benefit of using this agent.

However, there are several limitations to be pointed out. Most of the studies included in this meta-analysis are retrospective studies which are prone to biases. We are also limited in the number of sample size collected and are unable to further stratify the risk of ATTR cardiac amyloidosis patients based on their genetic mutations again due to the lack of results reported by each study. Finally, most patients included are in a stable heart failure class of NYHA I–II, hence, the benefit of diflunisal in the latter stage of disease progression is still unknown. Future prospective studies and randomized clinical trials will be needed to provide a definitive evidence regarding the role of diflunisal in treating cardiac amyloidosis.

## Conclusion

Diflunisal therapy is associated with a better survival benefit compared to the control group although it could be limited by the foreseeable adverse effects. For the time being, diflunisal could serve as an alternative to a much more expensive drugs in treating ATTR cardiac amyloidosis patients.

## Supplementary Information

Additional file 1.

## Data Availability

No datasets were generated or analyzed during the current study.

## List of abbreviations

ATTR-CA: Transthyretin cardiac amyloidosis
ATTRh: Transthyretin amyloidosis hereditary type
ATTRv: Transthyretin amyloidosis variant type
ATTR-wt: Transthyretin amyloidosis wild type
BNP: Brain natriuretic peptide
CI: Confidence interval
eGFR: Estimated glomerular filtration rate
FDA: Food drug administration
GI: Gastrointestinal
GLS: Global longitudinal strain
IVSD: Interventricular septal defect
LVEF: Left ventricular ejection fraction
MD: Mean difference
NSAID: Non-steroidal anti-inflammatory drug
NT-pro BNP: N terminal pro brain natriuretic peptide
NYHA: New York Heart Association
PWD: Posterior wall diameter
RR: Relative risk
TTR: Transthyretin

## References

[1] Berthelot E, Broussier A, Hittinger L, Donadio C, Rovani X, Salengro E et al (2023) Patients with cardiac amyloidosis are at a greater risk of mortality and hospital readmission after acute heart failure. ESC Hear Fail 10(3):2042–2050 10.1002/ehf2.14337 PMC10192232 37051755

[2] de Marneffe N, Dulgheru R, Ancion A, Moonen M, Lancellotti P (2022) Cardiac amyloidosis: a review of the literature. Acta Cardiol [Internet] 77(8):683–692 35852493 10.1080/00015385.2021.1992990

[3] Bhogal S, Ladia V, Sitwala P, Cook E, Bajaj K, Ramu V et al (2018) Cardiac amyloidosis: an updated review with emphasis on diagnosis and future directions. Curr Probl Cardiol [Internet] 43(1):10–34 29173805 10.1016/j.cpcardiol.2017.04.003

[4] Kazi DS, Bellows BK, Baron SJ, Shen C, Cohen DJ, Spertus JA et al (2020) Cost-effectiveness of tafamidis therapy for transthyretin amyloid cardiomyopathy. Circulation 141(15):1214–1224 32078382 10.1161/CIRCULATIONAHA.119.045093 PMC7156331

[5] Ibrahim M, Saint Croix GR, Lacy S, Fattouh M, Barillas-Lara MI, Behrooz L et al (2022) The use of diflunisal for transthyretin cardiac amyloidosis: a review. Heart Fail Rev 27(2):517–524 34272629 10.1007/s10741-021-10143-4

[6] Marques N, Azevedo O, Almeida AR, Bento D, Cruz I, Correia E et al (2020) Specific therapy for transthyretin cardiac amyloidosis: a systematic literature review and evidence-based recommendations. J Am Heart Assoc 9(19):e016614 32969287 10.1161/JAHA.120.016614 PMC7792401

[7] Peiró-Aventín B, Cabrera-Romero E, Mora-Ayestarán N, Domínguez F, González-López E, Garcia-Pavia P (2024) Safety and efficacy of diflunisal in transthyretin cardiac amyloidosis. Rev Esp Cardiol 77(5):426–435 38325700 10.1016/j.rec.2023.10.016

[8] Castaño A, Helmke S, Alvarez J, Delisle S, Maurer MS (2012) Diflunisal for ATTR cardiac amyloidosis. Congest Heart fail 18(6):315–319 22747647 10.1111/j.1751-7133.2012.00303.x PMC3727153

[9] Quarta CC, Ozer S, Whelan CJ, Fontana M, Rowczenio DM, Gilbertson JA et al (2015) Diflunisal therapy for cardiac ATTR amyloidosis: a longitudinal, prospective, single centre study. Orphanet J Rare Dis [Internet] 10:1–225603901

[10] Siddiqi OK, Mints YY, Berk JL, Connors L, Doros G, Gopal DM et al (2022) Diflunisal treatment is associated with improved survival for patients with early stage wild-type transthyretin (ATTR) amyloid cardiomyopathy: the Boston University Amyloidosis Center experience. Amyloid 29(2):71–78 35083944 10.1080/13506129.2021.2000388 PMC9258521

[11] Choi B, Lasica M, Hare J, Chong S, Strachan L, Hocking J, Ting S, Gibbs S (2020) 105 diflunisal is effective and affordable treatment in transthyretin cardiac amyloidosis (ATTR-CM)-but only half of patients can tolerate it. Heart, Lung Circ 29:S83. 10.1016/j.hlc.2020.09.112

[12] Choi B, Lasica M, Huynh N, Sirdesai S, Nagarethinam M, Ting S et al (2022) Diflunisal significantly improves survival of patients with transthyretin (TTR) amyloidosis cardiomyopathy (ATTR-CM); a retrospective analysis. Hear Lung Circ [Internet] 31:S79–S80. 10.1016/j.hlc.2022.06.080

[13] Gilad A, Joshi T, Mendelson L, Berk J, Sanchorawala V, Ruberg F et al (2021) Treating transthyretin amyloid cardiomyopathy: a Comparison of diflunisal and tafamidis. J Am Coll Cardiol [Internet] 77(18):3296. 10.1016/S0735-1097(21)04650-7

[14] Mints YY, Berk JL, Connors L, Doros G, Gopal DM, Kataria S, Lohrmann G, Pipilas AR, Ruberg FL, Siddiqi OK (2019) Diflunisal is associated with improved mortality in patients with wild-type attr cardiac amyloidosis: the bu amyloidosis center experience. Circulation 140(Supp_1):A13123

[15] Hanson JLS, Arvanitis M, Koch CM, Berk JL, Ruberg FL, Prokaeva T et al (2018) Use of serum transthyretin as a prognostic indicator and predictor of outcome in cardiac amyloid disease associated with wild-type transthyretin. Circ Hear Fail 11(2):1–19 10.1161/CIRCHEARTFAILURE.117.004000 PMC5819619 29449366

[16] Ikram A, Donnelly JP, Sperry BW, Samaras C, Valent J, Hanna M (2018) Diflunisal tolerability in transthyretin cardiac amyloidosis: a single center’s experience. Amyloid [Internet] 25(3):197–202 30388377 10.1080/13506129.2018.1519507

[17] Koyama J, Minamisawa M, Sekijima Y, Ikeda SI, Kozuka A, Ebisawa S, Miura T, Motoki H, Okada A, Izawa A, Ikeda U (2015) Left ventricular deformation and torsion assessed by speckle-tracking echocardiography in patients with mutated transthyretin-associated cardiac amyloidosis and the effect of diflunisal on myocardial function. IJC Heart Vasc 9:1–10 10.1016/j.ijcha.2015.07.010 PMC5497336 28785698

[18] Lohrmann G, Pipilas A, Mussinelli R, Gopal DM, Berk JL, Connors LH et al (2020) Stabilization of cardiac function with diflunisal in transthyretin (ATTR) cardiac amyloidosis. J Card Fail 26(9):753–759 31805416 10.1016/j.cardfail.2019.11.024 PMC7758872

[19] Rapezzi C, Merlini G, Quarta CC, Riva L, Longhi S, Leone O et al (2009) Systemic cardiac amyloidoses: disease profiles and clinical courses of the 3 main types. Circulation 120(13):1203–1212 19752327 10.1161/CIRCULATIONAHA.108.843334

[20] Gertz MA, Dispenzieri A, Sher T (2015) Pathophysiology and treatment of cardiac amyloidosis. Nat Rev Cardiol 12(2):91–102 25311231 10.1038/nrcardio.2014.165

[21] Berk JL, Suhr OB, Obici L, Sekijima Y, Zeldenrust SR, Yamashita T et al (2013) Repurposing diflunisal for familial amyloid polyneuropathy: a randomized clinical trial. JAMA 310(24):2658–2667 24368466 10.1001/jama.2013.283815 PMC4139164

[22] Connors LH, Sam F, Skinner M, Salinaro F, Sun F, Ruberg FL et al (2016) Heart failure resulting from age-related cardiac amyloid disease associated with wild-type transthyretin: a prospective. Obs Cohort Stud Circ 133(3):282–290 10.1161/CIRCULATIONAHA.115.018852 PMC4718760 26660282

[23] Elliott P, Drachman BM, Gottlieb SS, Hoffman JE, Hummel SL, Lenihan DJ, Ebede B, Gundapaneni B, Li B, Sultan MB, Shah SJ (2022) Long-term survival with tafamidis in patients with transthyretin amyloid cardiomyopathy. Circ Heart Fail 15(1):e008193 34923848 10.1161/CIRCHEARTFAILURE.120.008193 PMC8763250

[24] Damy T, Garcia-Pavia P, Hanna M, Judge DP, Merlini G, Gundapaneni B et al (2021) Efficacy and safety of tafamidis doses in the tafamidis in transthyretin cardiomyopathy clinical trial (ATTR-ACT) and long-term extension study. Eur J Heart Fail [Internet] 23(2):277–285. 10.1002/ejhf.2027 33070419 10.1002/ejhf.2027 PMC8048553

[25] Epstein M (2002) Non-steroidal anti-inflammatory drugs and the continuum of renal dysfunction. J Hypertens Suppl Off J Int Soc Hypertens 20(6):S17-23 12683423

[26] Bindu S, Mazumder S, Bandyopadhyay U (2020) Non-steroidal anti-inflammatory drugs (NSAIDs) and organ damage: a current perspective. Biochem Pharmacol 180:114147 32653589 10.1016/j.bcp.2020.114147 PMC7347500

[27] Page J, Henry D (2000) Consumption of NSAIDs and the development of congestive heart failure in elderly patients: an underrecognized public health problem. Arch Intern Med 160(6):777–784 10737277 10.1001/archinte.160.6.777

[28] Heidenreich PA, Bozkurt B, Aguilar D, Allen LA, Byun JJ, Colvin MM et al (2022) 2022 AHA/ACC/HFSA guideline for the management of heart failure: a report of the american college of cardiology/american heart association joint committee on clinical practice guidelines. Circulation [Internet] 145(18):e895-1032. 10.1161/CIR.0000000000001063 35363499 10.1161/CIR.0000000000001063

