# Supplementary material for: Efficacy and safety of diflunisal therapy in patients with transthyretin cardiac amyloidosis (ATTR-CA): a systematic review and meta-analysis
Source: Egypt Heart J. 2025 Mar 11;77:30. doi: 10.1186/s43044-025-00625-3 (PMC11896961; doi:10.1186/s43044-025-00625-3)

**SUPPLEMENTARY FILES**

**Supplementary 1.** Search strategy

(“amyloidosis” OR “amyloid” OR “cardiac amyloidosis” OR “cardiac amyloid” OR “amyloidosis cardiomyopathy” OR “amyloid cardiomyopathy” OR “ATTR” OR “ATTR amyloidosis” OR “ATTR cardiac amyloidosis” OR “ATTR amyloid” OR “transthyretin amyloidosis” OR “transthyretin cardiac amyloidosis” OR “transthyretin cardiac amyloid” OR “ATTR cardiomyopathy” OR “transthyretin cardiomyopathy” OR “transthyretin amyloidosis cardiomyopathy” OR “transthyretin amyloid cardiomyopathy”) AND (“diflunisal”)

**Supplementary 2.** Risk of bias assessment

**Supplementary 3.** Post treatment change of troponin I level with diflunisal therapy


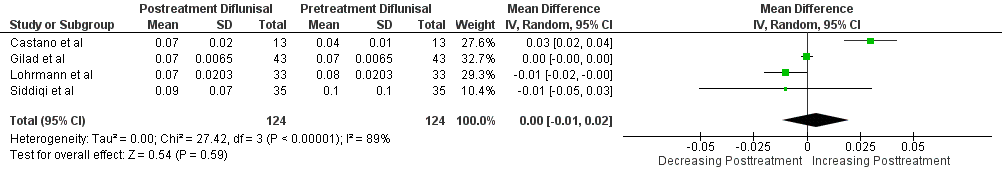


**Supplementary 4.** Post treatment change of E wave with diflunisal therapy


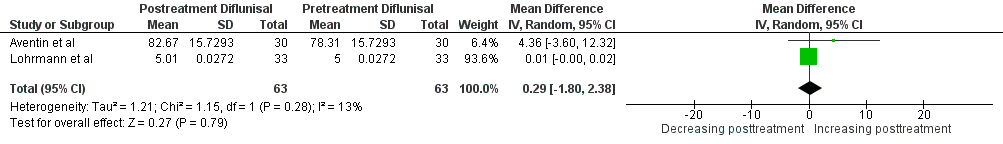


**Supplementary 5.** Post treatment change of BNP with diflunisal therapy


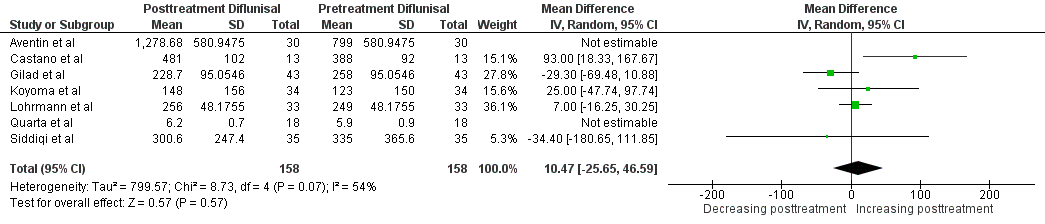


**Supplementary 6.** Post treatment change of posterior wall diameter with diflunisal therapy


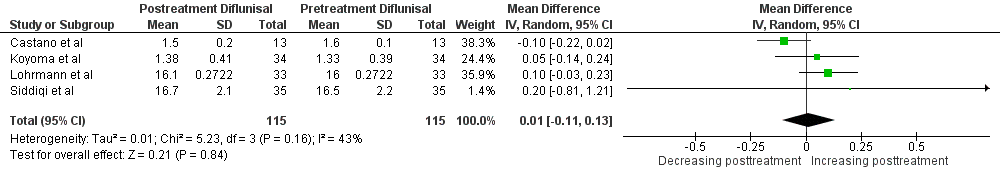


**Supplementary 7.** Post treatment change of intraventricular septum diameter with diflunisal therapy


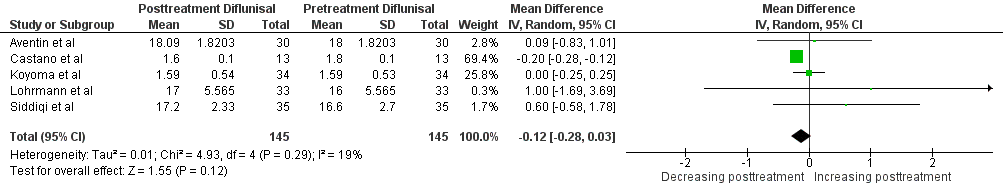


**Supplementary 8.** Post treatment change of global longitudinal strain with diflunisal therapy


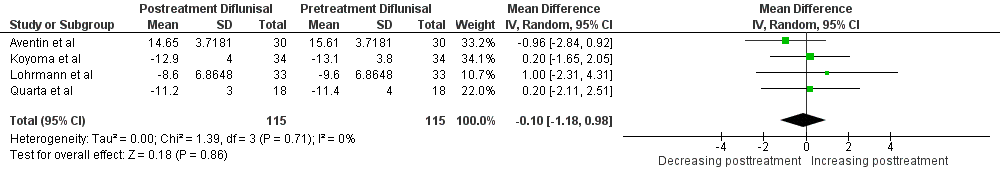


**Supplementary 9.** Post treatment change of left ventricular ejection fraction with diflunisal therapy
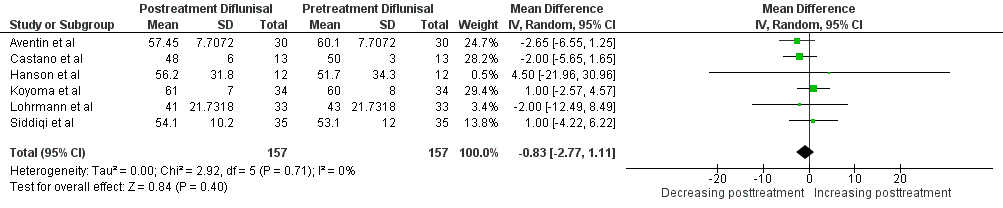


**Supplementary 11.** Change of BNP comparing diflunisal therapy with control


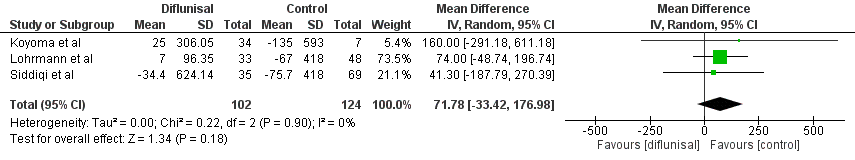


**Supplementary 12.** Change of E wave comparing diflunisal therapy with control


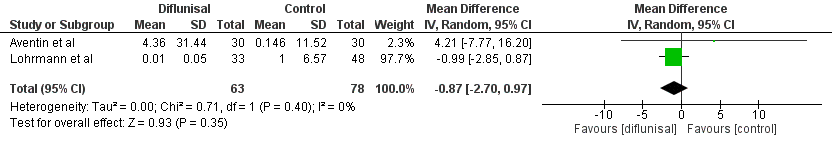


**Supplementary 13.** Change of GLS comparing diflunisal therapy with control


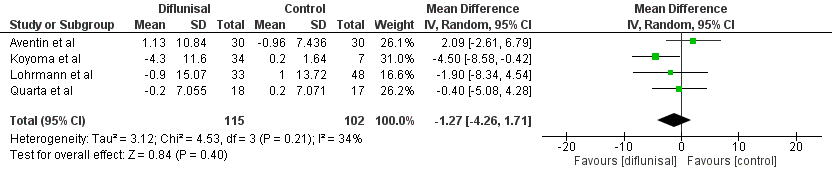


**Supplementary 14.** Change of IVSD comparing diflunisal therapy with control


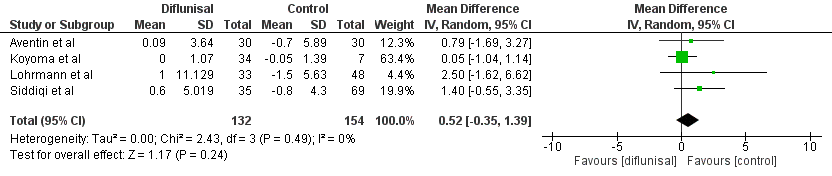


**Supplementary 15.** Change of LVEF comparing diflunisal therapy with control


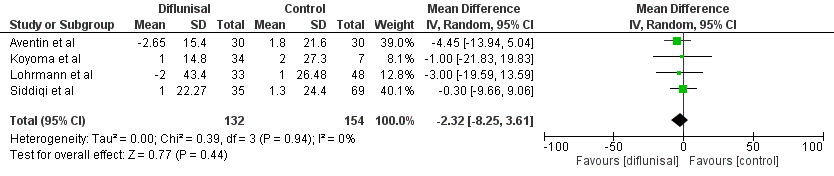


**Supplementary 16.** Change of PWD comparing diflunisal therapy with control


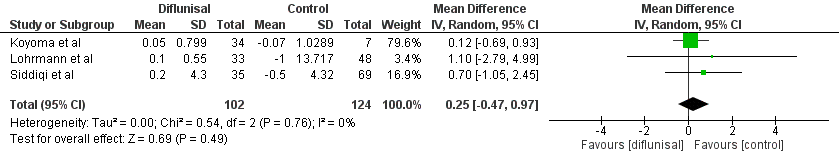


**Supplementary 17.** Reduction of mortality with diflunisal therapy after subgroup analysis of studies with 12 months follow up


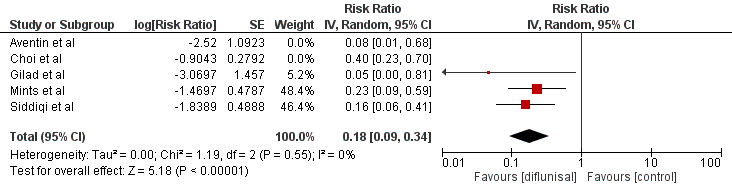


**Supplement 18**. Reduction of mortality with diflunisal therapy after subgroup analysis of studies of only multivariate analysis studies


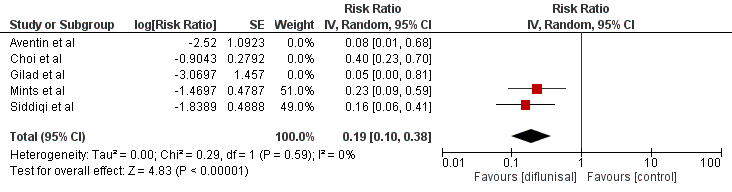


**Supplementary 19.** Posttreatment changes of creatinine with the use of diflunisal

**
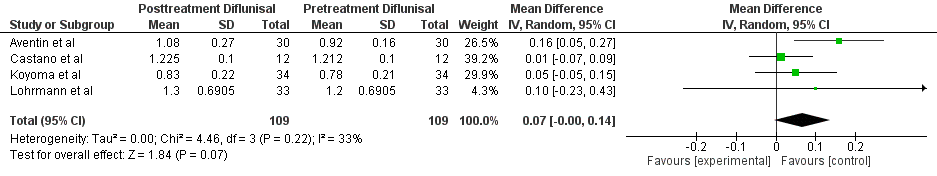
**

**Supplementary 20.** Posttreatment changes of eGFR with the use of diflunisal


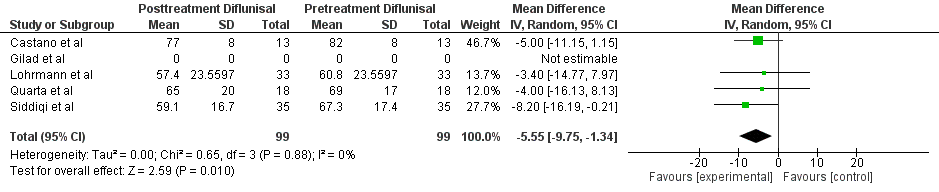


**Supplementary 21.** Posttreatment changes of hemoglobin with the use of diflunisal

**
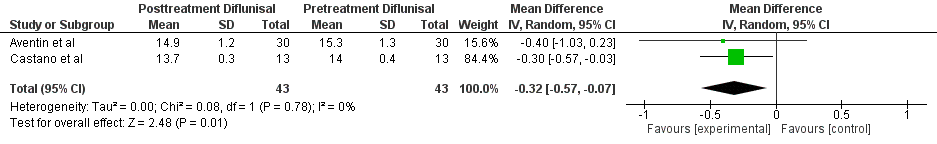
**

**Supplementary 22.** Posttreatment changes of platelet count with the use of diflunisal


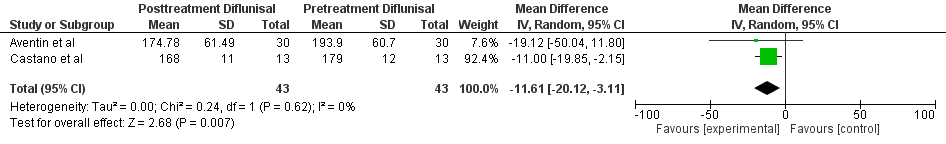

Supplement: Supplementary file 1 — Additional file 1. [file 43044_2025_625_MOESM1_ESM.docx]
